# Reactive oxygen species regulate urokinase plasminogen activator expression and cell invasion via mitogen-activated protein kinase pathways after treatment with hepatocyte growth factor in stomach cancer cells

**DOI:** 10.1186/1756-9966-28-73

**Published:** 2009-06-04

**Authors:** Kyung Hee Lee, Sang Woon Kim, Jae-Ryong Kim

**Affiliations:** 1 Department of Hematology-Oncology, College of Medicine, Yeungnam University, Daegu, Korea; 2 Surgery, College of Medicine, Yeungnam University, Daegu, Korea; 3 Biochemistry and Molecular Biology, College of Medicine, Yeungnam University, Daegu, Korea; 4 Aging-Associated Vascular Disease Research Center, College of Medicine, Yeungnam University, Daegu, Korea

## Abstract

**Background:**

Reactive oxygen species (ROS) are closely associated with the intracellular signal cascade, thus strongly implicating involvement in tumor progression. However, the mechanism by which ROS are generated and how ROS target downstream molecules to trigger tumor metastasis is unclear. In this study, we investigated the underlying signal pathways in ROS-induced urokinase plasminogen activator (uPA) expression in the human gastric cancer cells, NUGC-3 and MKN-28.

**Methods and Results:**

Intracellular ROS, as determined using the fluorescent probe, 2'-7' dichlorofluorescein diacetate, decreased after treatment with hepatocyte growth factor (HGF). We confirmed that Rac-1 regulated ROS production after activation of the AKT pathway with HGF. Exogenously added H_2_O_2 _promoted the expression of HGF, but not in a dose-dependent manner and also showed negative expression of HGF after co-treatment with H_2_O_2 _and HGF. Treatment with NAC, an intracellular free radical scavenger, decreased the enhancement of uPA production and tumor invasion in both cells. We clarified the downstream pathways regulated by ROS after treatment with H_2_O_2_, which showed negative control between FRK and p38 kinase activities for uPA regulation.

**Conclusion:**

HGF regulates Rac-1-induced ROS production through the Akt pathway and ROS regulates uPA production and invasion via MAP kinase, which provides novel insight into the mechanisms underlying the progression of gastric cancer.

## Background

Gastric cancer is the second most common cause of cancer death worldwide despite of the improved prognosis. To understand the precise mechanisms underlying invasion and metastasis would be helpful in improving survival. ROS, such as superoxide anion (O_2_^-^), hydrogen peroxide (H_2_O_2_), and hydroxyl radical (HO^-^), have emerged as highly toxic agents responsible for a wide variety of tissue damage [1] The involvement of these ROS in the pathogenesis of gastric diseases first became evident from the study of gastric mucosal injuries under normal conditions. ROS are relatively harmless, but when produced excessively or during deficient antioxidant defense, the oxidant and antioxidant balance is disturbed and the metabolites become toxic, which may lead to the initiation and promotion of cancer [2]. However, despite the positive correlation between the increased generation of ROS and the invasion of cancer, the specific mechanisms by which antioxidants act to suppress cancer development through ROS is unknown.

HGF has multiple biologic effects on a wide variety of cells, including mitogenic, motogenic, morphogenic, and anti-apoptotic activities [3,4]. The receptor for HGF is c-Met, a proto-oncogene product. Overexpression and mutation of the c-Met receptor has been well-described in various cancers [5,6]. Some studies have reported that HFG stimulates the migration and invasiveness of transformed epithelial cells concomitantly with the up-regulation of uPA [7]. In a separate study, HGF/c-Met signaling enhanced gastric cancer cell proliferation and increased uPA synthesis and activity. Inhibition of uPA receptors by monoclonal antibody against the uPA receptor decreased tumor cell invasion. Mitogen-activated protein kinase (MAPK) transduces extracellular signals into cellular responses, and thus plays an important role in proliferation, apoptosis, differentiation, and migration [8,9]. Gupta et al. [10] reported that increased ROS levels enhance MAP kinase activity for malignant progression of mouse keratinocyte cell lines.

In this study, we found that HGF modulates Rac-1-regulated ROS production, ROS induces the expression of uPA via the MAPK pathway, and stimulates the invasiveness of human gastric cancer cells.

## Methods

### Cell cultures

Two human gastric cancer cell lines (a poorly differentiated adenocarcinoma [NUGC-3] and a moderately differentiated tubular adenocarcinoma [MKN-28]), which were obtained from the Korea Cell Line Bank (Seoul, Korea), were used in the experiments described herein. Cells were maintained in Dulbecco's modified Eagle's medium (DMEM) supplemented with 10% fetal bovine serum, 1 mM sodium pyruvate, 0.1 mM non-essential amino acids, 2 mM L-glutamine, a 2-fold vitamin solution, and 50 U/ml penicillin/streptomycin (Life Technologies, Inc., Gaithersburg, MD, USA) in an incubator under a humidified atmosphere of 5% CO_2 _and 95% air at 37°C. Unless otherwise noted, cells were passaged and removed at 70% to 80% confluency.

### Reagents and antibodies

Antibodies against ERK, p38, phospho-ERK, and phospho-p38 were purchased from Cell Signaling Technology (Beverly, Massachusetts, USA). Antibodies against AKT, phosphor-AKT, and Rac1 were obtained from Santa Cruz Biotechnology, Inc. (Santa Cruz, California, USA). N-acetylcysteine (NAC), hydrogen peroxide (H_2_O_2_), and LY 294002 were purchased from Sigma (St. Louis, Missouri, USA). 2'-7'-dichlorofluorescin diacetate (DCF-DA) was obtained from Molecular Probes (Eugene, Oregon, USA). Horseradish peroxidase-conjugated anti-mouse and anti-rabbit antibodies were purchased from Bio-Rad Laboratories (Philadelphia, Pennsylvania, USA). Recombinant human HGF (R&D Systems, Inc, Minneapolis, Minnesota, USA) and human uPA antibody (389; American Diagnostica, Greenwich, Connecticut, USA) were also purchased. A dominant positive Rac-1 (Q61L) plasmid was kindly provided by Dr. K. Hahn of the university of North Carolina.

### Real-time PCR

Complementary DNA (cDNA) was synthesized from total RNA using MMLV reverse transcriptase (Promega Corp., Madison, Wisconsin, USA) by the oligo (dT) priming method in a 10 μl reaction mixture. Real-time PCR analysis was performed using a lightCycler1.5 Instrument (Roche, Mannheim, Germany). PCR was performed in a LightCycler capillary in a 10 μl reaction volume that contained 1* DNA Master SYBR Green I, 2.5 mM MgCI_2_, 1 μl cDNA, and 0.4 uM primers. The PCR protocol was as follows: initial denaturation for 2 minutes at 95°C, 45 cycles at 95°C for 10 seconds, 60°C for 5 seconds, and 72°C for 12 seconds. Results were analyzed with LightCycler Software, version 3.5.3. Sequence-specific primers for HGF were a forward primer, gggctgaaaagattggatca and a reverse primer, ttgtattggtgggtgcttca.

### Western blot analysis

Cells were harvested and incubated with a lysis buffer (50 mM Tris-HCl [pH 8.0], 150 mM NaCl, 1 mM EDTA, 1% Trion X-100, 10% glycerol, 1 mM PMSF, 1 mM sodium vanadate, and 5 mM NaF) with protease inhibitors and centrifuged at 15,000 rpm at 4°C for 10 min. Proteins (50 μg) were separated on 10% SDS-polyacrylamide gels and transferred to nitrocellulose membranes. The membranes were soaked with 5% non-fat dried milk in 10 mM Tris-HCl (pH 7.5), 150 mM NaCl, and 0.05% Tween-20 (TTBS) for 30 min and then incubated overnight with a primary antibody at 4°C. After washing 6 times with TTBS for 5 min, the membranes were incubated with a horseradish peroxidase-conjugated secondary antibody for 90 min at 4°C. The membranes were rinsed 3 times with TTBS for 30 min and the antigen-antibody complex was detected using the enhanced chemiluminescence detection system.

### Measurement of Rac-1 activity

Rac-1 activity was measured using the Rac-1 activation kit (Upstate Biotechnology, New York, USA). Briefly, whole-protein extracts were immunoprecipitated with the protein binding domain of PAK-1 PBD. PAK-1 PBD only binds to activated forms of Rac-1 and cdc42. Immunoprecipitated proteins were separated in SDS-polyacrylamide gels and blotted with anti-Racl.

### Measurement of ROS

ROS production was measured using the DCF-DA assay. In brief, cells were seeded in 60 mm culture dishes at 70% confluence and then starved in DMEM for 24 h. The cells were treated with HGF (0, 10, or 40 ng/ml). After treatment with HGF, cells were incubated with 10 μM of DCF-DA for 10 min. The cells were harvested, washed once, and resuspended in PBS. Fluorescence was monitored using a flow cytometer (Becton-Dickinson, San Jose, California, USA). The mean of the DCF fluorescence intensity was obtained from 10000 cells using 480 nm excitation and 540 nm emission settings. By using the same settings, the fluorescent intensity was obtained from each experimental group. Fluorescent levels were expressed as the percentage increase over the control.

### Standard two chamber invasion assay

Cells (1 × 10^4^) and NAC (5 mM) were placed in the upper chamber of a matrigel migration chamber with 0.8-micron pores (Fisher Scientific, Houston, TX, USA). Media containing 5% FBS and HGF (0 or 10 ng/mL), with or without NAC (5 mM), was added to the bottom chamber. After incubation for 48 hours, the cells were fixed and stained using a HEMA 3 stain set (Curtis Matheson Scientific, Houston, Texas, USA) according to the manufacturer's instruction. The stained filter membrane was cut and placed on a glass slide. The migrated cells were counted under light microscopy (10 fields at 200× power).

### Statistical analysis

The results of three independent experiments were expressed as the means ± SD and were analyzed by Student's *t*-test.

## Results

### HGF suppresses ROS generation in c-Met-overexpressing gastric cancer cells

**Figure 1:**
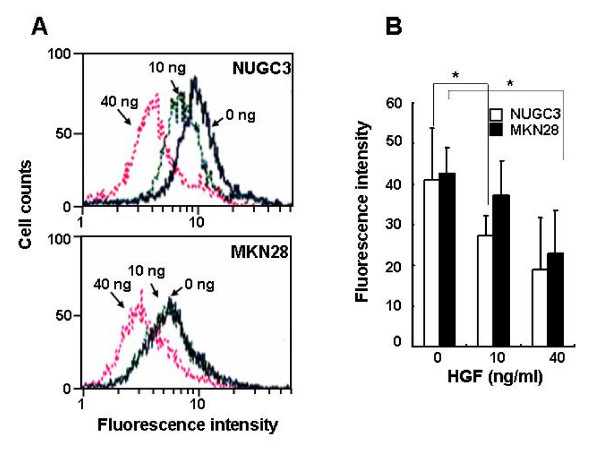
**Effects of HGF on ROS accumulation**. Serum-starved cells were treated with increasing concentrations of HGF (0, 10, and 40 ng/ml). After incubation for 1 h, the cells were incubated with DCF-DA (10 μM) for 10 min. The cells were washed with PBS, trypsinized, and resuspended in PBS. The intensity of DCF-fluorescence was immediately measured with a flow cytometer (A). Mean fluorescence intensity was obtained from 3 independent experiments and plotted (B). Representative data from 3 independent experiments were shown. Values are the means ± SD of three independent experiments. Statistical significance was estimated by Student's *t*-test (*, *p *< 0.05).

The intracellular ROS levels in c-Met-overexpressing NUGC-3 and MKN-28 cells treated with HGF were determined using DCF-DA by flow cytometry. Stimulation of c-Met-overexpressing gastric cancer cells with HGF significantly reduced the basal level of ROS in a dose-dependent manner (Figure 1).

### HGF suppresses Rac-1-regulated ROS production through activation of Akt

**Figure 2:**
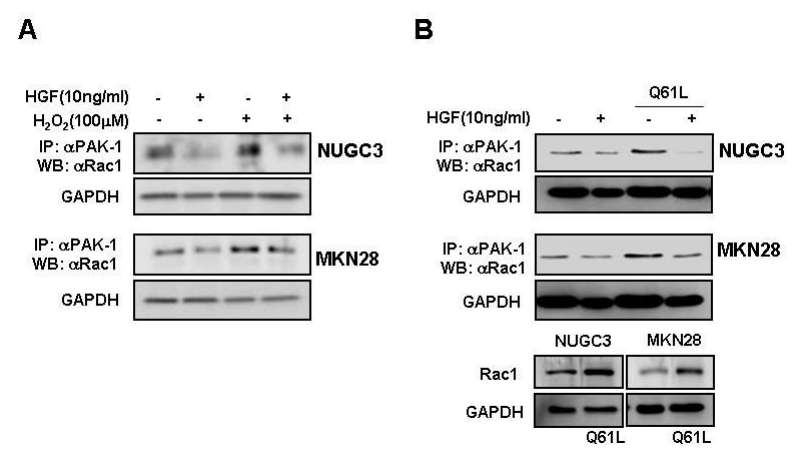
**Effects of HGF and H_2_O_2_/LY 294002 on Rac-1 activation**. Serum-starved cells was pretreated with or without H_2_O_2 _(100 μM) for 30 min and then treated with or without 10 ng/ml HGF (A). Rac-1 dominant positive cells (Q61L) were treated with or without HGF (B). After incubation for 15 min, the cells were collected, washed, and then sonicated. Cell lysates were immunoprecipitated with PAK-1 PBD and Rac-1 activation was measured by Western blotting with a Rac-1 antibody. Representative data from three independent experiments were shown.

**Figure 3:**
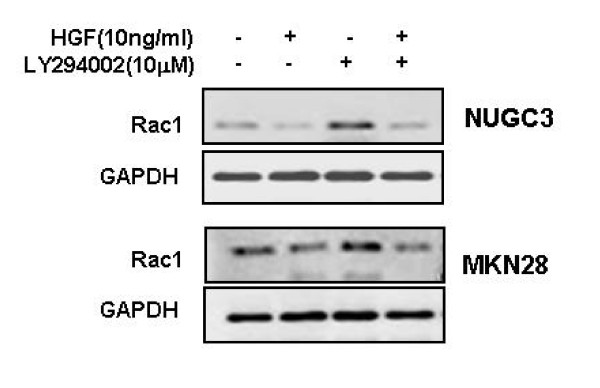
**Effects of HGF and LY 294002 on Rac-1 activation**. Serum-starved cells was pretreated with or without LY (10 μM) for 30 min and then treated with or without HGF. After incubation for 15 min, the cells were collected, washed, and then sonicated. Cell lysates were immunoprecipitated with PAK-1 PBD and Rac-1 activation was measured by Western blotting with a Rac-1 antibody. Representative data from three independent experiments were shown.

**Figure 4:**
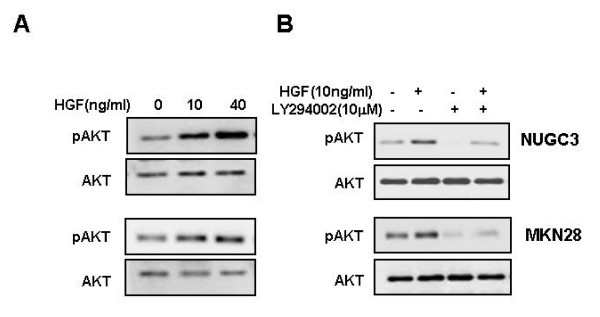
**Effects of HGF or LY 294002 on Akt phosphorylation**. Serum-starved cells were treated with increasing concentrations of HGF for 15 min. The protein levels of Akt and phospho-Akt were measured by Western blot analysis (A). Serum-starved cells were pretreated with LY 294002 (10 μM) for 30 min and then treated with HGF (10 ng/ml). After incubation for 15 min, the protein levels of Akt and phospho-Akt were determined by Western blot analysis (B). Representative data from three independent experiments are shown.

**Figure 5:**
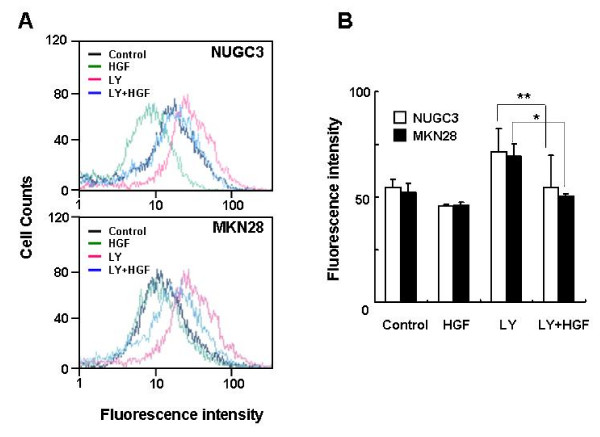
**Effects of LY 294002 on ROS accumulation**. Serum-starved cells were pretreated with LY 294002 (10 μM) for 30 min and then treated with HGF (10 ng/ml). The intensity of DCF fluorescence was measured with flow cytometry (A). Mean fluorescence intensity was obtained from 3 independent experiments and plotted (B). Representative data from three independent experiments are shown. Values are the means ± SD of three independent experiments. Statistical significance was estimated by Student's *t*-test (*, *p *< 0.05; **; *p *< 0.01).

We examined the role of HGF in modulating ROS production, particularly as regulated by Rac-1. Treatment with HGF suppressed the basal activity of Rac-1 and increased Rac-1 activity induced by H_2_O_2 _treatment (Figure 2A). In addition, treatment with HGF suppressed also the Rac-1 activity increased in Rac-1 dominant positive cells (Figure 2B). Pretreatment of cells with LY 294002, a PI3-kinase inhibitor, activated Rac-1 (Figure 3). Next, we examined whether Akt is involved in the reduction of the ROS level induced by HGF. Treatment of NUGC-3 and MKN-28 cells with HGF caused Akt activation in a dose-dependent manner (Figure 4A) and pre-incubation of cells with LY 294002 reduced HGF-induced Akt phosphorylation (Figure 4B). Furthermore, inhibition of Akt by LY 294002 treatment increased the ROS levels. More importantly, the effect of LY 294002 was abolished by HGF, as determined using DCF-DA by flow cytometry (Figure 5). These results suggest that PI3-kinase is an essential mediator through which HGF inhibits ROS generation.

### Upregulation of HGF mRNA levels in gastric cancer cell lines treated with H_2_O_2_

**Figure 6:**
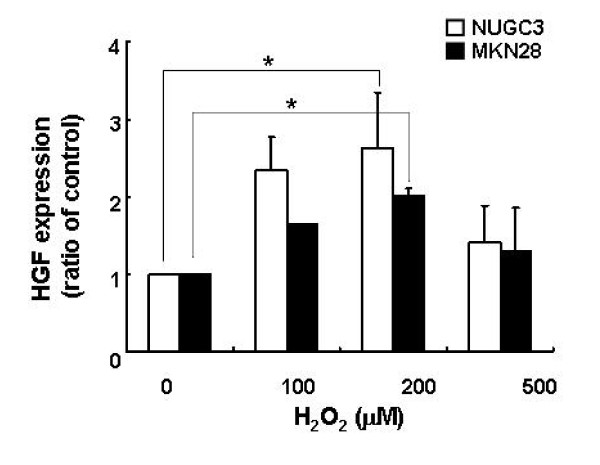
**Effects of H_2_O_2 _on the levels of HGF mRNA**. Cells were serum-starved and treated with increasing concentrations of H_2_O_2 _(0, 100, 200, and 500 μM). The expression level of HGF was measured by real-time RT-PCR. Values are the means ± SD of three independent experiments.

**Figure 7:**
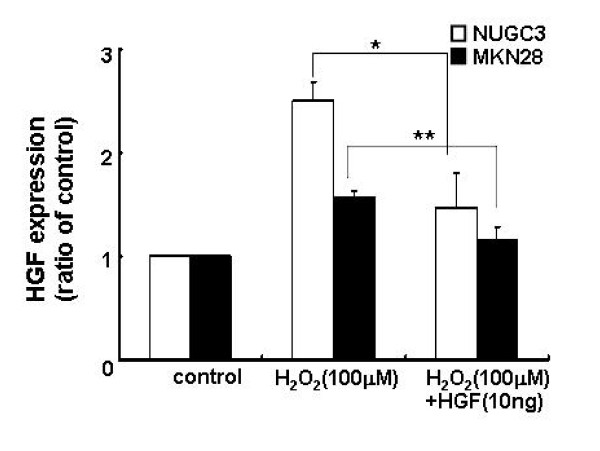
**Level of expression of HGF after treatment with H_2_O_2 _and/or HGF**. Cells were serum-starved and treated with H_2_O_2 _(100 μM) and/or HGF (10 ng/ml). The level of HGF mRNA was measured by real-time RT-PCR analysis. Values are the means ± SD of triplicates of three independent experiments. Statistical significance was estimated by Student's *t*-test (*, *p *< 0.05;**, *p *< 0.01).

To understand the mechanism of uPA production of ROS, we examined HGF gene expression using the RT-PCR method. The levels of HGF mRNA were 1.7–2.4 fold higher in cells treated with 100 μM H_2_O_2 _than in untreated cells. However, HGF mRNA levels were decreased when treated with 500 μM H_2_O_2 _(Figure 6). This might be due to H_2_O_2 _cytotoxicity. Subsequently, we measured HGF mRNA levels from both cell lines in the absence or presence of exogenous HGF and/or H_2_O_2_. The levels of HGF mRNA were suppressed by exogenous treatment of HGF and H_2_O_2 _(Figure 7).

### Effect of H_2_O_2 _and NAC on uPA production

**Figure 8:**
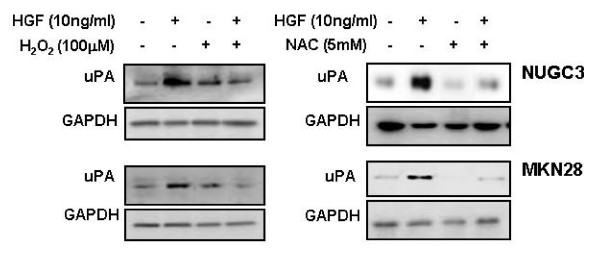
**Effects of H_2_O_2 _and NAC on HGF-mediated upregulation of uPA**. Serum-starved cells were pretreated with or without H_2_O_2 _(100 μM) and NAC (5 mM) for 30 min, and then treated with or without HGF (10 ng/ml). After incubation for 24 h, uPA secreted in culture media was measured by Western blot analysis with a uPA antibody. Representative data from 3 independent experiments were shown.

uPA gradually accumulated in both cell lines after treatment with HGF. Treatment with H_2_O_2 _resulted in an increase in the uPA protein level in both cell lines. When we treated cells with H_2_O_2 _and HGF, the uPA protein level was decreased. To investigate the effect of N-acetylcysteine (NAC), a precursor of glutathione and an intracellular free radical scavenger, on HGF-induced uPA production, we treated both cell lines with NAC. NAC decreased the HGF-induced uPA production (Figure 8).

### Effects of NAC on cell invasion

**Figure 9:**
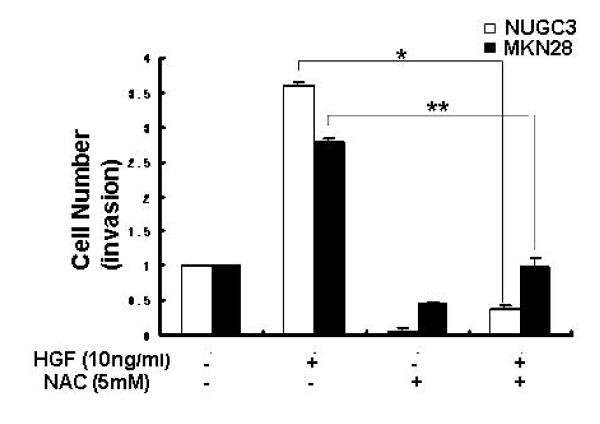
**Effects of NAC on *in vitro *invasiveness**. Cells in RPMI 1640 media supplemented with 5% FBS were placed in the upper chamber of Matrigel chamber and treated with or without NAC. The bottom chamber was filled with media containing 5% FBS and HGF with or without NAC. After 48 h of incubation, the cells which migrated through the filter were counted under light microscopy (10 fields at 200× power). Values are the means ± SD of triplicates of three independent experiments. Statistical significance was estimated by Student's t-test (*, *P *< 0.05; **, *p *< 0.01).

To examine the effects of HGF/c-Met-mediated uPA induction on the invasive properties of tumor cell phenotypes, we performed an *in vitro *invasion assay using a matrigal migration chamber. The invasiveness of HGF-treated cells was 2.7-fold higher in NUGC-3 cells, and 1.8-fold higher in the MKN-28 cells than in the untreated cells. We hypothesized that HGF-mediated uPA upregulation is responsible for the invasive properties of tumor cell phenotypes, and consequently blocking uPA activity could be a potential target in inhibiting tumor cell invasion. To test this hypothesis, we studied the effects of NAC on tumor cell invasiveness, and showed that invasiveness of NAC- treated cells was 50% lower in NUGC-3 cells and 90% lower in the MKN-28 cells than in the untreated cells. When we co-treated with exogenous HGF and NAC, cell invasion was also decreased. The fact that the invasive properties of these cells can be inhibited by NAC suggests that the regulation of ROS may be useful for a therapeutic target to halt metastasis in stomach cancers (Figure 9).

### Effect of H_2_O_2 _on ERK and p38 activation induced by HGF

**Figure 10:**
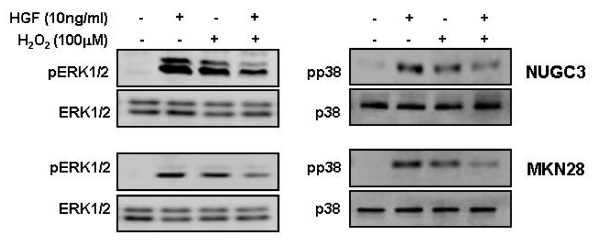
**Effects of H_2_O_2 _on ERK and p38 activation induced by HGF**. Serum-starved cells were pretreated with or without H_2_O_2 _(100 μM) for 30 min and then treated with or without HGF (10 ng/ml). After incubation for 15 min, the levels of phosphorylated ERK, ERK, phosphorylated p38, and p38 were measured by Western blot analysis. Representative data from 3 independent experiments are shown.

To demonstrate the effect of H_2_O_2 _on HGF-mediated ERK and p38 activation, we treated both cells with H_2_O_2_. Treatment with H_2_O_2 _increased the activity of ERK and p38. When cells were treated with H_2_O_2 _and HGF together, the activation of ERK and p38 kinase was decreased (Figure 10).

### Effect of ERK and p38 inhibitor on H_2_O_2_-induced uPA expression

**Figure 11:**
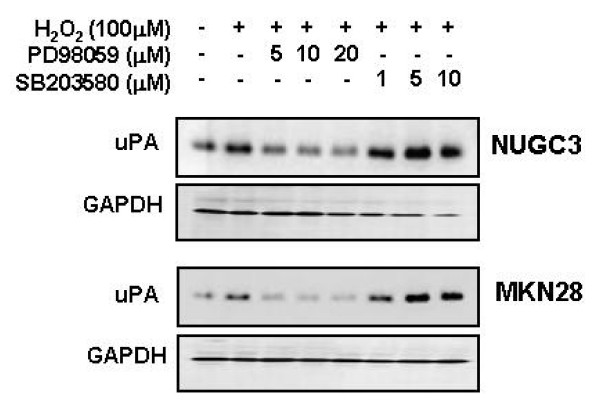
**Effects of PD 98059 or SB 203580 on HGF-mediated up-regulation of uPA**. Serum-starved cells were pretreated with or without H_2_O_2 _(100 μM) for 30 min and then treated with PD 98059 (5, 10 and 20 μM) or SB 203580 (1, 5 and 10 μM). After incubation for 24 h, uPA in culture media was measured by Western blot analysis. Representative data from 3 independent experiments were shown.

To test whether ERK and p38 activation was involved in H_2_O_2_-mediated uPA secretion, cells were pretreated with PD 098059 or SB 203580, and uPA secretion was measured by Western blotting. Both cells showed that H_2_O_2_-mediated uPA secretion was reduced with increasing concentrations of PD 098059. Densitometric analysis indicated that 10 μM PD 098059 reduced the urokinase secretion > 50%. In contrast, pretreatment with SB 203580 increased uPA secretion. These results suggested that H_2_O_2_-mediated uPA secretion and the augmentation of this activity was regulated by ERK and p38 activation (Figure 11).

### Effects of PD 098059 and/or SB 203580 on H_2_O_2_-induced ERK1/2 phosphorylation

**Figure 12:**
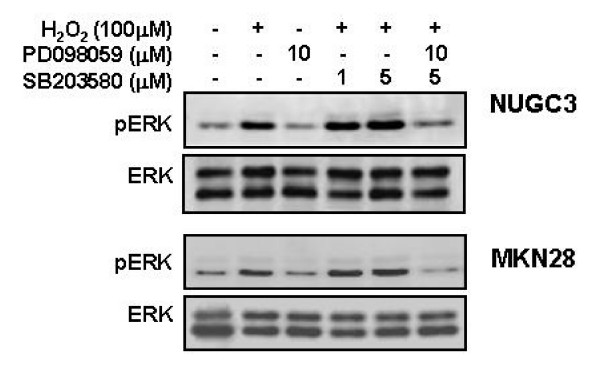
**Effects of PD 98059 and/or SB 203580 on H_2_O_2_-induced ERK phosphorylation**. Serum-starved cells were pretreated with PD 98059 (10 μM) and/or SB 203580 (1 and 5 μM) for 30 min and then treated with HGF (10 ng/ml) for 15 min. ERK activation was evaluated by Western blot analysis. Representative data from 3 independent experiments are shown.

To investigate the possibility of an interaction between ERK and p38 activation in H_2_O_2_-mediated uPA expression, the effect of SB 203580 on ERK activation was measured. Pretreatment with SB 203580 increased ERK phosphorylation in the H_2_O_2_-treated cells. Co-treatment with PD 098058 and SB 203580 decreased ERK phosphorylation. These results suggested that H_2_O_2_-mediated uPA secretion and the augmentation of this activity were regulated by ERK activation, and p38 activation might indirectly affect H_2_O_2_-mediated uPA secretion. In other words, the increments of H_2_O_2_-mediated uPA secretion and its level of expression according to the treatment by SB 203580 were mediated through ERK activation (Figure 12).

## Discussion

An abundance of evidence indicates the ROS play a central role in the key intracellualar signal transduction pathway for a variety of cellular process [11,12]. Aberrant ROS signaling may result in physiologic and pathologic changes, such as cell cycle progression [13], apoptosis, and aging [14]. Previously, elevated oxidative status has been found in many types of cancer cells, which contribute to carcinogenesis [15]. Recently, the involvement of ROS signaling in tumor metastasis was highlighted [16,17]. More evidence indicated that metastasis of tumor cells was closely associated with the microenvironment around the primary tumor lesions in which the growth factors and cytokines, such as transforming growth factor-β (TGF-β) and HGF, support malignant growth, invasion, and dissemination of the primary tumor [18]. Several important signal transduction pathways, such as MAPK, PI3K, and the Rho-GTPase cascades, are known to mediate transcriptional regulation of metastasis-related genes, such as MMPs [19]. Importantly, ROS are closely associated with these signal cascades, strongly implicating the involvement of ROS in tumor progression.

The Rac-1, a small GTPase, is an important regulator of ROS production within cells under hypoxia/re-oxygenation circumstances [20]. Rac-1 belongs to the rho family of small GTP-binding proteins and its role in the production of ROS in phagocytic cells, such as neutrophils, is well-established [21]. In such cells, Rac proteins are essential for the assembly of the plasma membrane NADPH oxidase, which is responsible for the transfer of electrons to molecular oxygen, leading to the production of superoxide anions. Rac-1-regulated ROS have been implicated in a variety of cellular process, including growth, migration, and transformation [22,23].

HGF is a prototypical prosurvival growth factor and also known to prevent non-transformed hepatocytes from oxidant-mediated apoptosis [24]. Ozaki et al. demonstrated that HGF-stimulated activation of pI3K-AKT is necessary and sufficient to suppress intracellular oxidative stress and apoptosis by inhibiting activation of pro-apoptotic, pro-oxidative Rac-1 GTPase [25]. In our study, we showed HFG decreased Rac-1 protein and ROS production, and intracellular ROS production increased when treated with the PI3-kinase inhibitor, LY294002. Miura et al have already reported that ROS promote rat ascites hepatoma cell invasion beneath mesentery-derived mesothelial cell monolayers. To investigate the mechanisms for this, they examined the involvement of HGF. The rat ascites hepatoma cell line, AH109A, expresses HGF and c-Met mRNAs. Treatment with ROS augments the amount of HGF mRNA in AH109A and the HGF concentration in the medium. ROS also induces HGF gene expression in mesothelial cells. Exogenously-added HGF enhances the invasive activity of AH109A cells. Pretreatment with ROS shows increased invasive activity, which is blocked by simultaneous pretreatment with anti-HGF antibody. These results suggest that the invasive activity of AH109A is mediated by autocrine and paracrine pathways of HGF, and ROS potentiate invasive activity by inducing gene expression of HGF in AH109A and mesothelial cells [26]. In our study, 100 μM H_2_O_2 _increased HGF gene expression. When we co-treated with exogenous HGF and H_2_O_2_, it showed downregulation of HGF gene expression.

The overexpression of uPA has been detected in various malignancies, including breast [27,28] and colon cancers [29]. Some dates have shown that a high level of uPA in tumors is associated with a rapid disease progression and a poor prognosis [30,31]. Miyazono et al. [32] showed oxidative stress induces uPA in RC-K8 human malignant lymphoma cells and H69 human small cell lung carcinoma cells. Kim et al. reported that ROS precedes the induction of uPAR expression, and this upregulation is attenuated by NAC, a ROS scavenger. In addition, exogenous ROS alone induced the expression and promoter activity of uPA [33]. Our study showed similar results with the above studies. Exogenous H_2_O_2 _increased uPA production and inhibited uPA after treatment of NAC.

Two of the candidate signaling molecules involved in EMT and cell migration are protein kinase C- (PKC) and MAP kinase-mediated signal pathways, which coordinate complex physiologic and pathologic events, including cell cycle control, differentiation, neo-angiogenesis, and metastasis [34]. Cytokines, such as TGF-β, HGF, and fibroblast growth factor, may stimulate tumor invasion metastasis via PKC and MAP kinase [35]. Mechanisms by which ROS affect signal transduction and gene expression have been described; some works have shown that ROS can activate MAPK, including ERK and p38 kinase. Meanwhile, serine and threonine protein kinase AKT is also regulated by exogenous and endogenous ROS [36,37]. How MAP kinase is activated by ROS to trigger cell migration is not clear. Protein kinase may be activated by ROS for a variety of cellular effects. Moreover, protein kinase is also an upstream kinase of MAPK required for cell migration [38]. Wu et al. [39] found ROS plays a central role in mediating PKC and ERK signaling for regulation of gene expression of integrins and E-cadherin that are responsible for EMT and migration of the human hepatoma cell line, HepG2. Lee et al. [40] examined the role of the MAP kinase signaling pathway on the stimulation of uPA synthesis in gastric cancer cells by using HGF. They showed that the phosphorylation of ERK and p38 kinase are dependent on the dosage of HGF and also clarified that uPA secretion and zymoactivity in the NUGC-3 cell lines were stimulated with HGF, which suggests the involvement of ERK and p38 kinase in the HGF-mediated uPA expression. The effects of PD098059 and SB203580 were measured in order to clarify which signaling pathway, between the ERK and p38 kinase pathways, plays the more important role in H_2_O_2_-induced uPA secretion. Increments of H_2_O_2_-mediated uPA expression via SB 203580 pretreatment were shown to be mediated by ERK activation, indicating that p38 kinase functions as a negative growth regulator. Xian et al. [41] also reported similar results in the PCNC-1 pancreatic cancer cell line.

**Figure 13:**
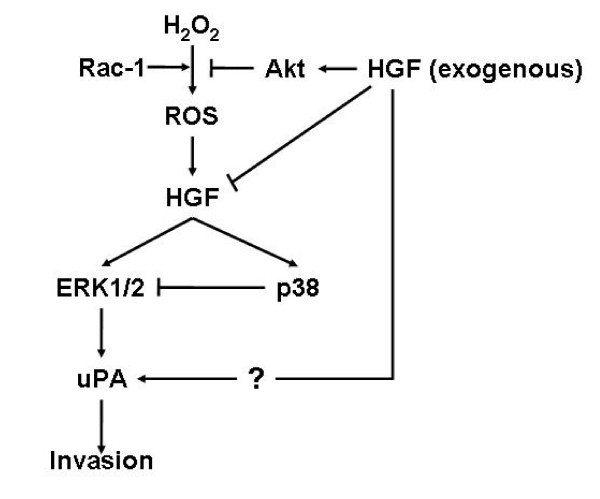
**Interaction of exogenous HGF with H_2_O_2 _in uPA expression**.

In this study, we showed that HGF decreased intracellular ROS and increased the uPA protein levels. Treatment with H_2_O_2 _also increased HGF mRNA and uPA protein. However, co-treatment with HGF and H_2_O_2 _decreased uPA, and HGF mRNA and protein levels increased by H_2_O_2 _treatment. These results suggest that exogenous HGF might play a negative role in the regulation of uPA protein levels increased by H_2_O_2 _treatment (Figure 13). Thus, further study is necessary to elucidate by which mechanism exogenous HGF regulates uPA protein levels through the regulation of intracellular ROS levels and signal pathways.

Overall, these results suggest that ROS are involved uPA regulation in control of tumor invasion and metastasis by cytokines, such as HGF in gastric cancer cells. Notwithstanding the above limitation, evidence that ROS directly contributes to HGF/c-Met-dependant tumor invasion and metastasis opens a novel perspective in the complex correlation between oxygen radicals and malignancy, and suggests new possibilities of antioxidant-based therapeutic intervention, complementary to the search for HGF/c-Met inhibitory compounds.

## Competing interests

The authors declare that they have no competing interests.

## Authors' contributions

KHL carried out cell treatment, cell transfection, immunoblotting analysis and drafted the manuscript. SWK participated in the design of the study, coordination and performed the statistical analysis. JRK supervised experimental work. All authors read and approved the final manuscript.
